# Regenerating Zebrafish Hearts Reveal the Molecular Agents of Repair

**DOI:** 10.1371/journal.pbio.0040281

**Published:** 2006-08-01

**Authors:** Liza Gross

Coronary disease has long been considered an affliction of the affluent, a lifestyle disease caused by eating unhealthy foods, drinking and smoking too much, and not getting enough exercise. But a recent study of the global burden of disease, published in *The Lancet*, found that heart disease is the number one killer in rich and poor countries alike. The disease is so deadly partly because the human heart, unlike the skin or liver, can't repair itself. Damaged heart tissue is replaced with scar tissue instead of healthy cells. Cardiomyocytes, the heart's structural cells, sometimes bulk up to replace lost cardiac cells, a response that can lead to cardiac arrest.

Not all hearts are so vulnerable to insults. In a 2002 study, Mark Keating and his colleagues showed that zebrafish, which can grow new spinal cords, retinas, and fins, can also regenerate heart tissue. And now, Ching-Ling Lien and colleagues, in a new study led by Keating, use microarray analysis to reveal the molecular signals underlying this regenerative capacity. The researchers identified sets of wound-healing genes and growth factors with temporally distinct expression patterns and show that regeneration relies on platelet-derived growth factor (PDGF), a regulator of cell proliferation and development.

In the 2002 study, Keating's group outlined the course of zebrafish heart regeneration. After an initial blood clot forms to stanch the bleeding, a more substantial fibrin clot forms a few days later. Nearby cardiomyocytes enter the cell cycle (triggering DNA synthesis and proliferation) about seven days after amputation to repair damaged heart tissue. By around 30 days, new cardiomyocytes have replaced lost tissue, and by two months, regeneration is complete.

To find potential drivers of heart regeneration, Lien et al. focused on the initial stages of regeneration. Following real and sham surgical amputations (the sham procedure stops just short of amputation to serve as a control), regenerating hearts were removed three, seven, and 14 days after the operation, and gene transcripts were extracted from the tissue. Microarray analysis revealed 662 transcripts with noticeably different expression patterns (increased or reduced activity) in at least one of the three time points.

The researchers classified the 662 genes into functional categories and expression patterns. Genes involved in the wound or inflammatory response were expressed early and peaked at three days, likely drawing inflammatory cells to the wound site. Starting from three days, several growth factors and secreted molecules, some associated with wound healing, also showed increased expression. By day 7, genes involved in tissue remodeling took over, with increased activity lasting a week.

By monitoring gene expression of a subset of growth factors and other secreted molecules within the regenerating heart, Lien et al. confirmed that their activity was restricted to specific times and sites in and around the wound and heart muscle. A crucial mediator of cell proliferation, PDGF-A had high expression levels at day 7. It often forms complexes with PDGF-B, but since the zebrafish version of the gene encoding this protein hadn't been sequenced yet, Lien et al. had to first isolate it from the tissue and sequence it before determining its expression pattern. As it happened, PDGF-B was also active at day 7—the same time that cardiomyocytes begin DNA replication. Working with cultured cardiomyocytes, the researchers demonstrated that PDGF signaling induces DNA synthesis in the cells and is required for cardiomyocyte proliferation during heart regeneration.

This study demonstrates the value of using microarrays to home in on likely mediators of regeneration at different stages of the process. With their list of candidate genes, Lien et al. plan to use more-targeted techniques to examine the genes' functions and to more fully investigate the role of PDGF signaling. And with the genetically pliable zebrafish as a model system, researchers will try to piece together the molecular program of heart regeneration, with an eye toward molecular therapies that could mend the human heart.

## 

**Figure:**
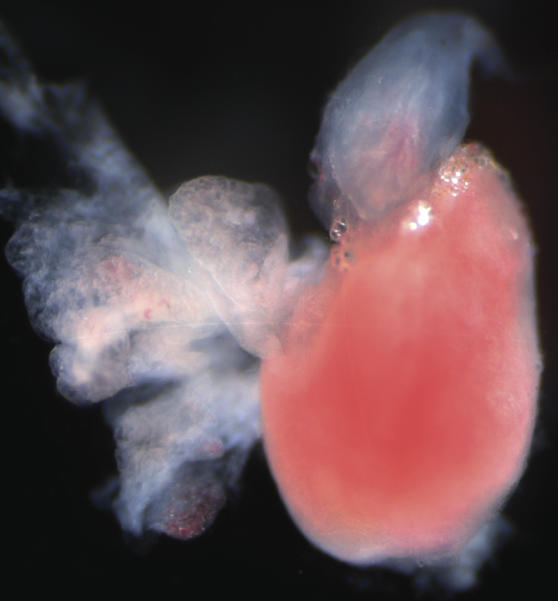
Gene expression profiling in a study of the mechanisms of zebrafish heart regeneration and cardiomyocyte proliferation uncovered secreted molecules that may play an important role in the regenerative process.

